# Hematological Malignancy Patients, COVID-19, and Favipiravir

**DOI:** 10.4274/tjh.galenos.2021.2021.0582

**Published:** 2021-12-07

**Authors:** Rujittika Mungmunpuntipantip, Viroj Wiwanitkit

**Affiliations:** 1 Private Academic Consultant, Bangkok, Thailand; 2 D.Y. Patil University, Pune, India

**Keywords:** Malignite, COVID-19, Favipiravir

## To the Editor,

We would like to share our ideas on the “Clinical Characteristics and Outcomes of COVID-19 in Turkish Hematological Malignancy Patients.” As Civriz Bozdağ et al. [1] noted in that study: “Treatments with hydroxychloroquine alone or in combination with azithromycin were associated with a higher rate of mortality in comparison to favipiravir use”. First, favipiravir is an antiviral drug proposed for management of COVID-19. Compared to other alternative drugs, including hydroxychloroquine and azithromycin, favipiravir should be more specifically applied in the management of viral infections. To determine the exact usefulness of favipiravir, clinical trials and observational studies are needed for assessment of drug efficacy and safety. Comparative studies between favipiravir and other therapies would also be useful and would yield more data on favipiravir. The report cited here is a good example of this. Another example is a previous comparative study of favipiravir therapy and other antiviral drugs [2].

Regarding the effects of favipiravir, some additional considerations should be discussed. Other concurrent therapies, such as steroid medications, can also affect the clinical outcome [3]. Additional analysis of the possible effects of concurrent use of steroids with favipiravir, hydroxychloroquine, azithromycin, or their combination would be interesting. Additionally, whether the efficacy of the drug is associated with specific types and severities of hematological malignancies is a point for further research. The day of initiation of favipiravir treatment is also an important factor for therapeutic outcome [4]. According to a recent report by Doi et al. [5], early and late administrations of favipiravir result in different clinical outcomes. More data on the exact period between onset of disease and start of favipiravir are required. Regarding the present report [1], Civriz Bozdağ et al. [1] might consider additional factor analysis regarding the time effect for assessment of the effect of favipiravir. If future study designs are to be based on the current report of Civriz Bozdağ et al. [1], it may be necessary to conduct an additional prospective study from the onset of COVID-19 to the start of favipiravir administration.
