# Supplementary material for: Hematological Malignancy Patients, COVID-19, and Favipiravir
Source: Turk J Haematol. 2021 Dec 7;38(4):331–2. doi: 10.4274/tjh.galenos.2021.2021.0582 (PMC8656123; doi:10.4274/tjh.galenos.2021.2021.0582)
Supplement: Related texts

# REPLY FROM THE AUTHORS

In our study, we reported the clinical characteristics and outcomes of 340 adult and pediatric COVID-19 patients with hematological malignancies from March to November 2020. The aim of the study was to evaluate the factors related to severity and mortality of COVID-19 retrospectively; the study was not statistically designed to show the impact of treatment on outcomes. There was no approved treatment for COVID-19 so treatment plans in our country entailed hydroxychloroquine (HCQ) alone or in combination with azithromycin as initial treatment and favipiravir for further progress of pneumonia in follow-up. In the later months, favipiravir was introduced to the initial treatment schedule for COVID-19, which resulted in the formation of different treatment groups within our retrospective data.

In multivariate analysis related to mortality, besides hematological disease status, decreased life expectancy related to primary hematological disease, neutropenia, and admission to the intensive care unit, the type of COVID-19 treatment was a significant factor. Patients treated with HCQ alone had 4.9-fold higher mortality risk in comparison to patients treated with favipiravir alone, while those treated with HCQ plus favipiravir had 2.0-fold risk and those treated with HCQ plus azithromycin had 2.1-fold risk. Favipiravir is an antiviral drug so a greater impact may be expected in comparison to HCQ. However, it should be kept in mind that the treatment policy in our country was changed after the negative results published on HCQ treatment. Furthermore, we did not have any other patients treated with different antiviral agents in our study. For patients treated with favipiravir as the initial treatment, all were treated with the drug on the day of COVID-19 diagnosis, so we do not have a second group for a comparison of the impact of the timeline, unfortunately. Anticytokine treatments were administered based on individual centers’ policies so the data were heterogenous for further analyses. We agree that prospective studies would better show the outcome differences between favipiravir and other antiviral therapies.

**Sinem Civriz Bozdağ, Güldane Cengiz Seval, İpek Yönal Hindilerden, Fehmi Hindilerden, Neslihan Andıç, Mustafa Baydar, Lale Aydın Kaynar, Selami Koçak Toprak, Hasan Sami Göksoy, Berrin Balık Aydın, Ufuk Demirci, Ferda Can, Vildan Özkocaman, Eren Gündüz, Zeynep Tuğba Güven, Zübeyde Nur Özkurt, Sinan Demircioğlu, Meral Beksaç, İdris İnce, Umut Yılmaz, Hilal Eroğlu Küçükdiler, Elgün Abishov, Boran Yavuz, Ünal Ataş, Yaşa Gül Mutlu, Volkan Baş, Fahir Özkalemkaş, Hava Üsküdar Teke, Vildan Gürsoy, Serhat Çelik, Rafiye Çiftçiler, Münci Yağcı, Pervin Topçuoğlu, Özcan Çeneli, Hamza Abbasov, Cem Selim, Muhlis Cem Ar, Orhan Kemal Yücel, Sevil Sadri, Canan Albayrak, Ahmet Muzaffer Demir, Nil Güler, Muzaffer Keklik, Hatice Terzi, Ali Doğan, Zeynep Arzu Yegin, Meltem Kurt Yüksel, Soğol Sadri, İrfan Yavaşoğlu, Hüseyin Saffet Beköz, Tekin Aksu, Senem Maral, Veysel Erol, Leylagül Kaynar, Osman İlhan, Ali Zahit Bolaman, Ömür Gökmen Sevindik, Arzu Akyay, Muhit Özcan, Günhan Gürman, Şule Ünal Cangül, Yasemin Yavuz, Reyhan Diz Küçükkaya, Güner Hayri Özsan**
